# Supplementary material for: Association between oral microbiome and seven types of cancers in East Asian population: a two-sample Mendelian randomization analysis
Source: Front Mol Biosci. 2023 Nov 21;10:1327893. doi: 10.3389/fmolb.2023.1327893 (PMC10702768; doi:10.3389/fmolb.2023.1327893)
Supplement: Supplementary file 1 [file DataSheet1.ZIP › Supplementary Materials/MR plots for tongue/tongue═╝/Colorectal cancer/pheno.866_to_colorectal cancer_forest.pdf]

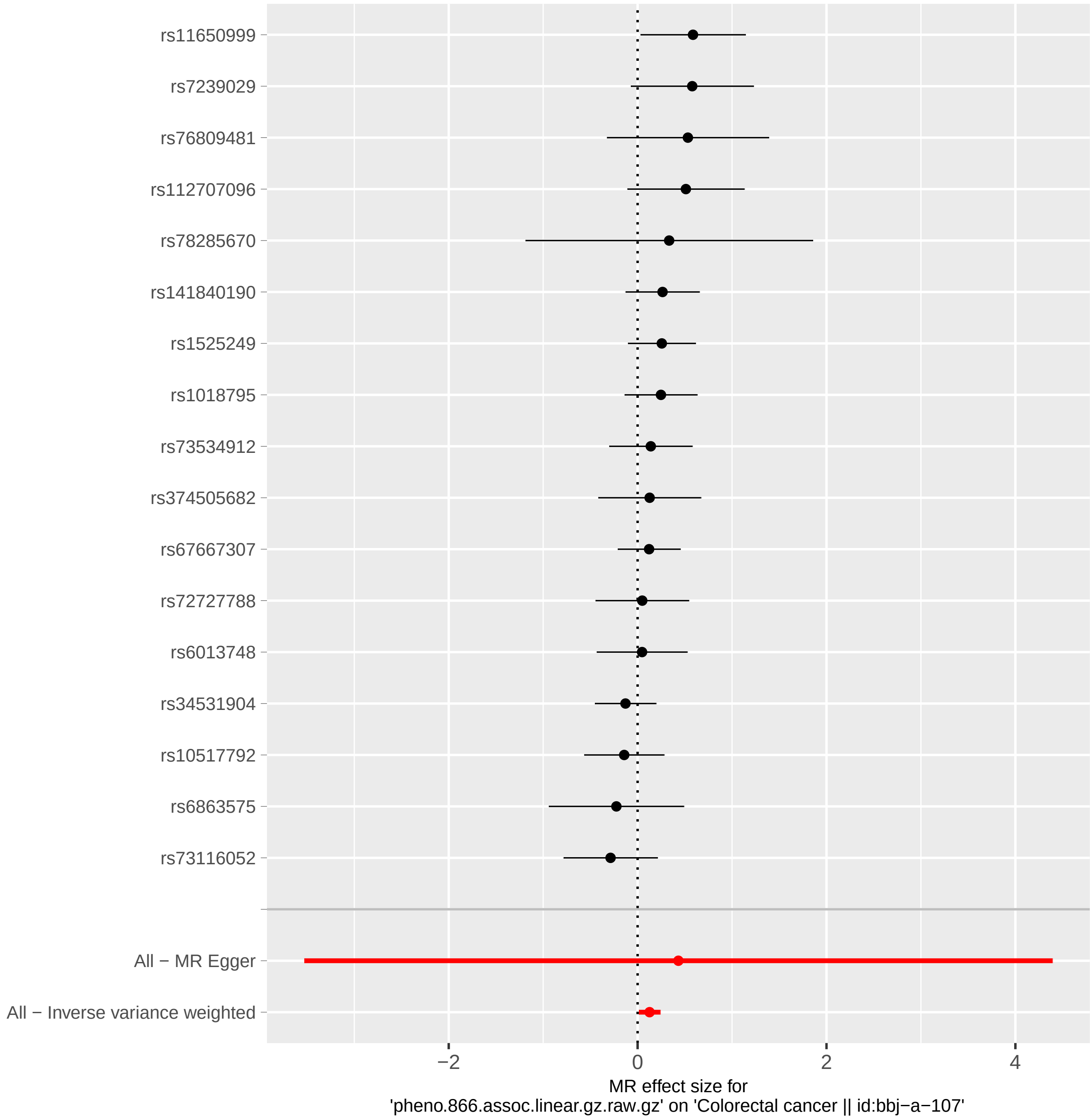
rs11650999
rs7239029
rs76809481
rs112707096
rs78285670
rs141840190
rs1525249
rs1018795
rs73534912
rs374505682
rs67667307
rs72727788
rs6013748
rs34531904
rs10517792
rs6863575
rs73116052
All − MR Egger
All − Inverse variance weighted
−2
0
2
4
MR effect size for
'pheno.866.assoc.linear.gz.raw.gz' on 'Colorectal cancer || id:bbj−a−107'
